# Mapping the Chemodiversity, Antioxidant and Enzyme Inhibitory Potential and *In Silico* Studies of *Heliotropium europaeum*

**DOI:** 10.1002/fsn3.70119

**Published:** 2025-04-01

**Authors:** Shabnam Mustafa, Muhammad Imran Tousif, Naheed Raiz, Muhammad Saleem, Saba Tauseef, Gokhan Zengin, Laiba Hassan, Areeba Hassan, Mamona Nazir, Shabbir Muhammad

**Affiliations:** ^1^ Institute of Chemistry, Baghdad‐ul‐Jadeed Campus The Islamia University of Bahawalpur Bahawalpur Pakistan; ^2^ Division of Science and Technology, Department of Chemistry University of Education Lahore Pakistan; ^3^ Dr. Panjwani Center for Molecular Medicine and Drug Research, International Center for Chemical and Biological Sciences University of Karachi Karachi Pakistan; ^4^ Department of Biology, Science Faculty Selcuk University Konya Turkey; ^5^ Department of Pharmacy Khwaja Fareed Campus, The Islamia University of Bahawalpur Bahawalpur Pakistan; ^6^ Department of Bioinformatics, Institute of Biochemistry, Biotechnology & Bioinformatics, Baghdad Campus The Islamia University of Bahawalpur Bahawalpur Pakistan; ^7^ Department of Chemistry Government Sadiq Women College University Bahawalpur Bahawalpur Pakistan; ^8^ Department of Chemistry, College of Science King Khalid University Abha Saudi Arabia

**Keywords:** antioxidant activities, docking studies, enzyme inhibition studies, *Heliotropium europaeum*, total bioactive contents, UHPLC–MS/MS analysis

## Abstract

The annual plant *Heliotropium europaeum* grows in waste ground next to agricultural land and along roadsides from June to September. This plant is indigenous to Southern and Central Europe, as well as Western Asia and Northern Africa; the term “Europaeum” most likely refers to the continent of Europe. According to a literature search, *H. europaeum* is used externally to cure warts and to aid in wound healing. It also has antibacterial, antifungal, antitumor, anti‐inflammatory, insecticidal, antispasmodic, cholagogue, emmenagogue, antipyretic, and anthelmintic properties. In the present investigation, we measured the biological activities of the extracts of *H. europaeum,* identification of secondary metabolites by UHPLC–MS/MS, and bioinformatics like docking studies against enzymes as evidence for the bioactivities. The results of chemical profiling showed that the HE‐M fraction had the highest phenolic and flavonoid contents (15.63 ± 0.39 mg GAE/g extract and 5.67 ± 0.03 mg QE/g extract, respectively), while the HE‐E fraction came in second (13.73 ± 0.35 mg GAE/g extract and 4.38 ± 0.08 mg QE/g extract, respectively). Similarly, out of all the extracts, the methanolic extract was found to have the highest antioxidant activity in DPPH and ABTS antioxidant assays, while the HE‐E extract was found active among all the extracts in all other antioxidant assays. In the enzyme inhibition assay, we had mixed results, where the HE‐E fraction was active against all the enzymes. The chemical profiling through UHPLC–MS/MS analysis endorsed the presence of many known pyrrolizidine alkaloids, flavonoids, and phenolic compounds, while the results of docking studies against the tested enzyme revealed the inhibitory action of pyrrolizidine alkaloids, flavonoids, and phenolic compounds. Further multivariate analysis was employed to find the relationship between total bioactive components and biological activities. This study established a foundation for utilizing these results and compounds as drug leads and for exploring the industrial applications of *H. europaeum* in developing products aimed at non‐communicable diseases.

## Graphical abstract

This study established a foundation for utilizing these results and compounds as drug leads ‐ for exploring the industrial applications of *Heliotropium europaeum* in developing products aimed at non‐communicable diseases.

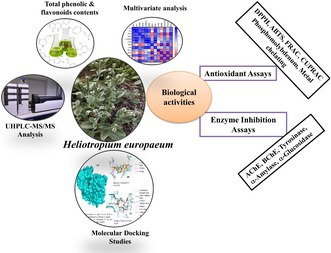

## 1 Introduction

Throughout the human civilization, plants have always been a vital source of food, shelter, transportation, and medicine. It is known that ancient man chewed on certain herbs to relieve pain or covered wounds with plant leaves to improve healing. It was a time when natural products were the sole means to treat diseases and injuries. However, new drug discovery has always been a challenge to scientific task to discover viable lead candidates. Drug discovery is a process that flows from the screening of natural products to a new isolate, and of course, it requires expertise and experience and is high‐cost process. However, now high‐throughput methods have revolutionized the screening of natural products and the discovery of new drugs in a time‐and cost‐effective way (Tariq and Siddiqi 1985).

Nature has decorated our world with a rich wealth of medicinal herbs, where every plant possesses its own therapeutic properties owing to its bioactive secondary metabolites. Plant properties to inhibit diseases make them extremely useful as natural drugs, because they provide small molecules having medicinal properties and low side effects (Koparde et al. 2019). Literature search disclosed that the raw materials for folk medicines are mostly coming from plant sources in the form of plant powders or their extracts; therefore, plants are a rich heritage of ethnobotanical usage (Kar 2003). More than ~75,000 higher plants exist on the earth, but only ~10% have been used in traditional medicine, with only ~5% have been explored scientifically for their therapeutic value (Calixto et al. 1998; Rao and Gurjar 1990). Therefore, there is a continuous need to investigate further plant sources for their chemodiversity and pharmacological actions, and to provide health care products to the increasing population around the globe.

*Heliotropium* of the Boraginaceae family is a genus comprised of about 250–300 species worldwide (Fayed 2021). The word “*Heliotropium*” is derived from the Latin word “Helios” meaning sun and “trope” meaning turning to, because its flowers usually turn to the sun (Pandey et al. 1996). *Heliotropium* plants are of local importance in folk medicine and possess several medicinal properties such as antibacterial and antifungal activities (Paulraj et al. 2013). However, some *Heliotropium* plants are reported as toxic because of the presence of pyrrolizidine alkaloids, which are shown to be responsible for many liver diseases (Fayed 2021). *Heliotropium* species are phytochemically bioactive and have remarkable healing effects (Fayed 2021). Previous studies on the biological and pharmacological activities of various parts of *Heliotropium* species have resulted in the discovery of bioactive compounds with anti‐bacterial, anti‐fungal, anti‐viral, anti‐tumor, antioxidant, anti‐inflammatory, anti‐platelet, wound healing, cardiotonic, contraceptive, and prostaglandin activities (Noumedem et al. 2013; Sharma et al. 2009); in addition, they also possess anti‐dysentery and anti‐diarrheal (Erosa‐Rejón et al. 2009) properties.

*Heliotropium europaeum* is an annual plant growing during June–September in waste ground near arable land and roadsides. The word “Europaeum” is probably related to the continent of Europe because this plant is native to Southern and Central Europe; in addition, it is also known to be native to western Asia and Northern Africa (Pandey et al. 1996) Literature search revealed that *H. europaeum* possesses antibacterial, antifungal, antitumor, anti‐inflammatory, insecticidal, antispasmodic, cholagogue, emmenagogue, antipyretic, and anthelmintic activities and is used externally to treat warts and to promote wound healing (Bonet and Valles 2007; Mahmood et al. 2011; Qureshi et al. 2010; Saeedi and Morteza‐Semnani 2009; Zargari 1990). This plant produces pyrrolizidine alkaloids, including heliotrine, heleurine, supinine, europine, and lasiocarpine. The hepatatoxic effects of *H. europaeum* are attributed to these alkaloids (Duke 2002; Qureshi et al. 2010). It is also reported that *H. europaeum* causes poisoning in sheep when they ingest food contaminated with seeds of this plant (Al‐Snafi 2018; Pass et al. 1979; Shimshoni et al. 2015). In addition, few reports are also found about *H. europaeum* causing poisoning in humans due to herbal teas contaminated with seeds of this plant; these reports declare the seeds of *H. europaeum* are rich in hepatotxic pyrrolizidine alkaloids (Diaz 2015; Elvin‐Lewis 2005; Fu et al. 2017; Pass et al. 1979; Wiedenfeld and Edgar 2011). About 30 pyrrolizidine alkaloids are reported in *H. europaeum* (Shimshoni et al. 2015). Some literature reports on other *Heliotropium* species have emphasized that species of this genus are non‐toxic (Khider et al. 2020). Although previous Phytochemical analysis of *H. europaeum* indicated the presence of alkaloids, terpenoids, steroids, saponins, flavonoids, phenols, and tannins (Duke 2002; Mukhtar et al. 2023; Saeedi and Morteza‐Semnani 2009), most of these reports focused on the toxic properties of this plant; whereas other bioactive compounds and their important biological activities were ignored. However, Al‐Saleem et al. isolated kaempferol, luteolin, quercetin, kaempferol‐3‐O‐glucoside, lutin‐7‐O‐glucoside, caffeic acid, rosmarinic acid, and methyl rosmarinate and have studied their antioxidant, lipoxygenase (LOX)‐ and angiotensin‐converting enzyme (ACE) inhibitory activities; in addition, the report emphasizes the beneficial effects of *H. europaeum* (Al‐Saleem et al. 2020).

Keeping in view the above contradictory reports on the medicinal properties and uses of *H. europaeum* , we decided to investigate its chemodiversity and further medicinal potential of this plant. Therefore, in the present study, we evaluated various extracts of the aerial parts of *H. europaeum* (without seeds and fruits) for its phenolic and flavonoid contents, total antioxidant capacity through DPPH, ABTS, CUPRAC, FRAC, phosphomolybdenum, and metal chelating assays. In addition, its anti‐Alzheimer's disease, anti‐diabetic, and skin care properties have also been studied through enzyme inhibition assays. The pharmacological potential of *H. europaeum* was also substantiated through molecular docking studies against various enzymes.

## 2 Materials and Methods

### 2.1 Plant Material and Extraction

In May 2023, aerial parts of the *Heliotropium europaeum* plant were collected from the desert and identified by Dr. Farrukh Nisar, a plant taxonomist at the Department of Biochemistry, Cholistan University of Veterinary and Animal Sciences, Bahawalpur. a voucher specimen with the number HE‐3‐5‐2023 was deposited in the herbarium of the same university. The plant material (5.0 kg) was chopped into small pieces, dried for 15 days in the shade, then macerated twice for 7 days using 8.0 L of distilled methanol. A dark green sticky extract (HE‐M, 110 g) was obtained by evaporating the solvent with a rotary evaporator at 40 C° with 280 rounds per minute. The HE‐M (100 g) of extract was suspended in distilled water (500 mL) and extracted using n‐hexane and ethyl acetate to obtain 30 g of soluble fractions of hexane (HE‐H) and 25 g of soluble fractions of ethyl acetate (HE‐E), respectively. The aqueous layer (HE‐W, 40 g) was dried to obtain it.

### 2.2 Determination of Total Phenolic and Flavonoid Contents

The Folin–Ciocalteu procedure was used to quantify the total phenolic contents (TPC), and aluminium chloride tests were used to estimate the total flavonoid contents (TFC). (Slinkard and Singleton 1977; Tousif et al. 2022) In these analyses, flavonoid and phenolic contents are expressed as rutin (mg RE/g extract) and gallic acid (mg GAEs/g extract) equivalents, respectively.

### 2.3 Antioxidant Assays

Already established protocols (Grochowski et al. 2017; Tousif et al. 2024) were followed to estimate the antioxidant activity of the extracts. FRAP, ABTS, DPPH, CUPRAC, and total antioxidant capacity were expressed as trolox equivalents, while ethylene diamine tetraacetic acid (EDTA) was used as a reference for the metal chelating assay (Saleem et al. 2021; Tousif et al. 2022).

### 2.4 Enzyme Inhibition Assays

Acetylcholinesterase (AChE), Butyrylcholinesterase BChE, tyrosinase, α‐amylase, and α‐glucosidase enzyme inhibitory assays were also performed according to reported methods in the literature (Mollica et al. 2017; Tousif et al. 2023; Zubair et al. 2022) AChE and BChE inhibitory activity was measured as an equivalent of galantamine (mg GALAE/g extract), α‐amylase and α‐glucosidase inhibitory potential as an equivalent of acarbose (mmol ACAE/g extract), while for tyrosinase activity, kojic acid (mmol KAE/g extract) was used as the reference drug.

### 2.5 Multivariate Analysis

The experiments were performed in triplicate, and differences between the extracts were compared using an ANOVA and Tukey's test. Pearson correlation analysis was used to establish the link between total bioactive components and biological activity assays. Graph Pad Prism (version 9.2) was used for the analysis. To assess the degree of similarity or difference between the extracts, a PCA was carried out using SIMCA (version 14.0).

### 2.6 UHPLC–MS/MS Experiments

Chemical profiling of methanolic plant extract was unveiled through ultra‐high performance liquid chromatography mass spectrometry (UHPLC–MS/MS) analysis on an Agilent 1290 Infinity UHPLC system coupled to an Agilent 6520 Accurate‐Mass Q‐TOF mass spectrometer with a dual ESI source as reported previously (Mustafa et al. 2023; Saleem et al. 2021).

### 2.7 Docking Studies

The 3D sdf structural data for the compounds were obtained through Pubchem (Kim et al. 2019). The compounds were converted to pdb format as described previously (Tousif et al. 2024). To obtain the protein structures, the Protein Data Bank (PDB) database was used (Sussman et al. 1998). To ascertain the drug‐target interaction, the compounds were molecularly docked to BChE, AChE, α‐glucosidase, α‐amylase, and tyrosinase using Autodock software (version 4.2) (Morris et al. 2009). The ligands were allowed to become flexible while the receptor proteins were made rigid in order to orient and investigate the most likely binding pose. The previously documented docking protocol was adhered to for docking and for post‐dock analysis of the binding poses obtained after docking analysis (Saleem et al. 2023).

## 3 Results and Discussion

### 3.1 Determination of Various Classes of Secondary Metabolites

Phytochemical screening of all the extracts of *H. europaeum* showed the presence of various bioactive secondary metabolites (Table 1). Flavonoids and glycosides were missing in the HE‐H fraction, while the water fraction (HE‐W) comprises phenolics and glycosides, while other metabolites were missing in this fraction. The HE‐E and HE‐M contain all types of secondary metabolites except that terpenoids were absent in the HE‐E. Alkaloids are the major class of Heliotropium plants, and all fractions of *H. europaeum* displayed their presence except the HE‐W fraction (Table 1). It is worth noting that usually alkaloids are extracted with water only if they are present as salts, like hydrochlorides. In addition, terpenoids and steroids were also absent in HE‐W, whereas only phenolics were detected in all the fractions (Table 1). This analysis revealed that *H. europaeum* is rich in a variety of important bioactive components.

**TABLE 1:** Chemical analysis of all the extracts of *H. europaeum*.

Extract/Fraction	Alkaloid^a^	Phenolics^b^	Flavonoids^c^	Terpenoids^d^	Steroids^d^	Glycosides^e^
HE‐M	+	+	+	+	+	+
HE‐H	+	+	−	+	+	−
HE‐E	+	+	+	−	+	+
HE‐W	−	+	−	−	−	+

^a^ Mayer's and Dragendroff's tests.

^b^ Ferric chloride test.

^c^ NaOH, Zinc/HCl and lead acetate tests.

^d^ Salkowski's test.

^e^ Keller‐kiliani test. + = detected; − = not detected.

### 3.2 Estimation of Total Phenolic and Flavonoid Contents

All the extracts of *H. europaeum* were screened for their phenolic (TPC, gallic acid equivalent) and flavonoid (TFC, quercetin equivalent) contents (Table 2). The HE‐M contains maximum phenolic and flavonoid contents with values of 15.63 ± 0.39 mg GAE/g extract and 5.67 ± 0.03 mg QE/g extract, respectively, whereas the HE‐E fraction was second in number (13.73 ± 0.35 mg GAE/g extract and 4.38 ± 0.08 mg QE/g extract, respectively). Although HE‐H showed some phenolic content but flavonoids were absent in this fraction (Table 2). Usually, flavonoids are relatively polar phenolic compounds until they have multiple methoxyl groups in their structures; therefore, there are fewer chances for the flavonoids to be extracted with a non‐polar solvent like hexane, whereas phenolic acids in the form of their alkyl esters can be extracted in hexane. Therefore, it can be predicted that phenolics present in the HE‐H fraction can be the low polar esters of phenolic acids. HE‐W also constitutes reasonable phenolic contents; however, it was poor in flavonoids. Previously reported data on phenolic and flavonoid contents of *Heliotropium* plants substantiated our results, with the highest phenolic and flavonoid contents in the methanolic fraction (Qayyum et al. 2016; Wasiullah et al. 2019).

**TABLE 2:** Phenolics (TPC) and flavonoids (TFC) in the extracts/fractions of *H. europaeum*.

Extract/Fractions	Total phenolic contents (mg GAE/g extract)	Total flavonoid contents (mg QE/g extract)
HE‐M	15.63 ± 0.39	5.67 ± 0.03
HE‐H	11.79 ± 0.22	Not Detected
HE‐E	13.73 ± 0.35	4.38 ± 0.08
HE‐W	12.40 ± 0.04	0.38 ± 0.02

Phenolic compounds are a the main class of secondary metabolites in plants, which are generally involved in defense against ultraviolet radiation or belligerence by pathogens (Beckman 2000) and protect plants from environmental stress. These metabolites are divided into phenolic acids, flavonoids, and other polyphenols and are found in free form or combined with mono‐ and polysaccharides, or can occur as derivatives, such as esters (Harborne et al. 1985). Phenolics may also contribute to the bitterness, astringency, color, flavor, odor, and oxidative stability of the plant (Pandey and Rizvi 2009). In terms of their medicinal values, it is an established fact that continuous intake of herbs rich in phenolics provides protection against the development of cancers, cardiovascular diseases, diabetes, osteoporosis, and neurodegenerative diseases (Arts and Hollman 2005; Graf et al. 2005). This could be the reason that there is an interest in using food supplements and other nutraceuticals comprising phenolic compounds has been increased during the last decade. In fact, the free radical scavenging properties of phenolics help to prevent various chronic and oxidative stress‐related diseases (Caleja et al. 2017; Vuong and Atherton 2017). Since *H. europaeum* is rich in a variety of phenolics, it can also be a component in nutraceutical formulations if its toxicity is studied.

### 3.3 Chemical Profiling by UHPLC–MS/MS Analysis

The chemical profiling of the methanolic extract of *H. europaeum* was performed through UHPLC–MS/MS (Table 5), which resulted in the identification of 91 metabolites. The identified compounds include phenolics, flavonoids, alkaloids, and terpenoids. The previous investigation on *H. europaeum* reported the identification of eight phenolic compounds: kaempferol, luteolin, quercetin, kaempferol 3‐O‐glucoside, luteolin 7‐O‐glucoside, in addition to caffeic acid, rosmarinic acid, and methyl rosmarinate, which possess potent antioxidant, lipoxygenase, and angiotensin‐converting enzyme activity. Among the identified compounds the quercetin possessed the most potent antioxidant and ACE inhibitory activity with (IC_50_ = 8.1 and 17.5 μM, respectively), whereas rosmarinic acid and its methyl ester showed the strongest LOX‐inhibitory activity (IC_50_ = 4.2 and 3.6 μM, respectively) (Al‐Saleem et al. 2020). In another study, quantification of phenolic compounds was conducted and the majority of the phenolic components found in the methanol extract of *H. europaeum* were epicatechin (7243.33 μg/g), caffeic acid (15971.41 μg/g), 2,5‐dihydroxybenzoic acid (11469.11 μg/g), and quercetin (4465.59 μg/g) (Ozay 2023).

### 3.4 Antioxidant Activities of the Extracts of *H. europaeum*

#### 3.4.1 Free Radical Scavenging Activities

DPPH and ABTS free radical scavenging activities were measured as trolox equivalent for all the extracts. HE‐M and HE‐W were nearly equally active against DPPH free radical with values of 3.78 ± 0.87 and 3.71 ± 0.66 mg TE/g extract, but HE‐H and HE‐E were found inactive in this test (Table 3). However, in the ABTS assay, all the extracts exhibited significant activities, with the highest value (35.762 ± 1.77 mg TE/g extract) attributed to the HE‐M fraction, followed by the HE‐E and HE‐W (Table 3) fractions; with the lowest associated with HE‐H (29.90 ± 0.96 mg TE/g extract). These results disclosed a correlation between phenolic contents and free radical scavenging activity.

**TABLE 3:** Antioxidant activities of the extracts/fractions of *H. europaeum*.

Extract/Fractions	DPPH (mg TE/g extract)	ABTS (mg TE/g extract)	CUPRAC (mg TE/g extract)	FRAP (mg TE/g extract)	Phosphomolybdenum (mmol TE/g extract)	Metal chelating (mg EDTAE/g extract)
HE‐M	3.78 ± 0.87	35.762 ± 1.77	76.78 ± 1.68	29.74 ± 0.21	1.38 ± 0.012	10.88 ± 0.67
HE‐H	na	29.90 ± 0.96	34.45 ± 0.43	22.79 ± 0.22	0.57 ± 0.0	29.74 ± 0.17
HE‐E	3.52 ± 0.85	32.27 ± 2.29	83.88 ± 2.45	35.45 ± 0.72	1.48 ± 0.07	30.05 ± 0.19
HE‐W	3.71 ± 0.66	31.5 ± 1.15	30.07 ± 0.12	19.16 ± 0.14	0.49 ± 0.03	8.55 ± 0.83

Literature reports revealed that several *Heliotropium* plant extracts (whole plant or part of plant) or pure compounds (phenolics or flavonoids) have shown varying degrees of DPPH and ABTS free radical scavenging activities (Ahmad et al. 2016; Aïssaoui et al. 2019; Fayed 2021; Karakaya et al. 2019) with the polar fractions exhibiting higher potential (Hussain et al. 2010; Ozntamar‐Pouloglou et al. 2023), which substantiate our results. Furthermore, our previous study on *Heliotropium crispum* collected from the same habitat also showed similar results (Arshad et al. 2021), thus endorsing our present study. Additionally, the methanol extract demonstrated the highest antioxidant activity in another study that tested the antioxidant potencies of the extracts (ethanol, methanol, chloroform, and water) using six distinct antioxidant assays: metal chelating, reducing power (FRAP), phosphomolybdenum, β‐carotene/linoleic acid assay, and radical quenching (ABTS and DPPH) (Ozay 2023) which also verifies our results.

#### 3.4.2 Antioxidant Activities in CUPRAC and FRAP Assays

The chemical diversity of antioxidants makes it difficult to separate and quantify antioxidants from the vegetable matrix. CUPRAC and FRAP methods are based on an electron‐transfer mechanism and are considered the most reliable methods to measure the total antioxidant capacity of a herbal extract (Apak et al. 2008a, 2008b, 2004; Özyürek et al. 2011). Specifically, the CUPRAC method has been reported to be more advantageous over FRAP, and certain phenolics like epicatechin gallate, epigallocatechin gallate, quercetin, fisetin, epigallocatechin, catechin, and caffeic acid are reported to possess the highest reducing power in this assay; it is further added that the cupric ion reducing property is attributed to the number and position of ‐OH groups in a phenolic molecule as well as the level of conjugation (Apak et al. 2008a). Apak et al. have further established that phenolic compounds, through their free hydroxyl groups, reduce cupric ionsbetter, thus imparting potential antioxidant properties to the plant extracts rich in phenolics and flavonoids (Apak et al. 2008b).

All the extracts of *H. europaeum* were tested for their antioxidant potential in CUPRAC and FRAP assays. In both the assays, the HE‐E fraction exhibited the highest reducing potential with values of 83.88 ± 2.45 and 35.45 ± 0.72 mg TE/g extract, respectively (Table 3), followed by the HE‐M fraction, which showed inhibitory potential with the values of 76.78 ± 1.68 and 29.74 ± 0.21 mg TE/g extract, respectively. Since the hexane extract (HE‐H) showed the least activity, it substantiated that phenolic and flavonoid content are responsible for the metal reducing potential of *H. europaeum* . A moderate metal reducing power of the HE‐H fraction (Table 3) could be attributed to metabolites other than phenolic compounds, probably having non‐phenolic hydroxyl groups or some low polar phenolic esters. Other *Heliotropium* plant extracts have also been reported to possess comparable activity in FRAP and CUPRAC assays (Ozntamar‐Pouloglou et al. 2023). The present new study disclosed overall significant antioxidant potential of *H. europaeum* , which was also supported by our previous study on another *Heliotropium* plant (Arshad et al. 2021) and thus makes it a candidate for further study as part of nutraceuticals.

#### 3.4.3 Total Antioxidant Capacity Measurement in Phosphomolybdenum Assay

All the extracts of *H. europaeum* were subjected to total antioxidant capacity measurement by the phosphomolybdenum method, which is based on the reduction of Mo(VI) to Mo(V) indicated by the formation of a green phosphate complex at acidic pH (Elkhamlichia et al. 2017). The HE‐E fraction showed the highest antioxidant capacity (1.48 ± 0.07 mmol TE/g extract) followed by HE‐M (1.38 ± 0.012 mmol TE/g extract), whereas HE‐H and HE‐W were least active (Table 3) in this test. These observations and the results from the above antioxidant assays were all proportional to the higher total phenolic and flavonoid contents in the respective extracts. Our present results are in full agreement with our previous studies (Ali et al. 2021; Khan et al. 2019) and other literature reports, which also showed a linear relationship between total phenolic and flavonoid contents and antioxidant activity of the plant extracts (Diwan et al. 2012; Kasangana et al. 2015).

Metal chelating activity of all the extracts of *H. europaeum* was also measured as equivalent to EDTA contents, where HE‐E and HE‐H showed almost equal potential (30.05 ± 0.19 and 29.74 ± 0.17 mg EDTAE/g extract, respectively); meanwhile, polar fractions HE‐M and HE‐W were found least active (Table 3) in this test. Ozntamar‐Pouloglou et al. studied extracts of *Heliotropium procubens* (Ozntamar‐Pouloglou et al. 2023), where almost similar potential has been reported, thus substantiating our findings. Further, our observations revealed that secondary metabolites other than phenolics may also contribute to chelating activities. Since *Heliotropium* plants also contain a high amount of alkaloids, these compounds may impart metal chelating activity to the extracts.

### 3.5 Enzyme Inhibitory Activities

#### 3.5.1 AChE And BChE Inhibition Activities

All the extracts of *H. europaeum* were also evaluated for their acetylcholinesterase (AChE) and butyrylcholinesterase (BChE) inhibitory activities as galantamine equivalent (Table 4). HE‐M, HE‐E, and HE‐W fractions displayed nearly equal potential with values of 4.12 ± 0.06, 4.00 ± 0.02, and 4.16 ± 0.13 mg GALAE/g extract, respectively, while HE‐H exhibited slightly lower but significant inhibitory activity of AChE enzyme inhibition. However, against BChE, the HE‐H fraction was the most active (5.05 ± 0.12 mg GALAE/g extract) followed by the activities of HE‐E (4.39 ± 0.49 mg GALAE/g extract), HE‐M (2.67 ± 0.14 mg GALAE/g extract) and HE‐W (1.95 ± 0.26 mg GALAE/g extract). In previous studies, it is found that the crude extract of *Heliotropium procubens* exhibited weaker activities (Ozntamar‐Pouloglou et al. 2023), while *Heliotropium crispum* collected from the same habitat as *H. europaeum* showed similar potential (Arshad et al. 2021), which could be attributed to different metabolic profiles due to different environmental conditions. Further, the activity trend in the present study revealed that a decrease in polarity of the extracting solvent increases the activity, indicating that instead of phenolics, other components could have more impact on the cholinesterases inhibitory activities.

**TABLE 4:** Enzyme Inhibition activities of the extracts/fractions of *H. europaeum*.

Extract/Fractions	AChE (mg GALAE/g extract)	BChE (mg GALAE/g extract)	Tyrosinae (mg KAE/g extract)	α‐Amylase (mmol ACAE/g extract)	α‐Glucosidase (mmol ACAE/g extract)
HE‐M	4.12 ± 0.06	2.67 ± 0.14	47.63 ± 1.43	0.26 ± 0.00	1.77 ± 0.00
HE‐H	3.87 ± 0.32	5.05 ± 0.12	41.40 ± 1.15	0.43 ± 0.01	Inactive
HE‐E	4.00 ± 0.02	4.39 ± 0.49	47.16 ± 0.90	0.42 ± 0.01	1.77 ± 0.04
HE‐W	4.16 ± 0.13	1.95 ± 0.26	47.79 ± 2.90	0.18 ± 0.02	1.71 ± 0.02

**TABLE 5:** UHPLC–MS/MS analysis of the methanolic extract of *H. europaeum*.

Sr. No.	Analyte Peak Name	Retention Time	Area/Height	Formula Mass	Precursor Mass	Identified Compound	Class of compound	Mol. Formula	Fragments
1	337.1240 (M + Na^+^)	4.36	5.66	314.1353	337.126	9,10‐Epoxy‐18‐hydroxystearate	Fatty acid	C_18_H_34_O_4_	82, 112, 142, 155, 245
2	414.2445	6.68	5.10	413.2377	414.244	β‐sitosterol	Steroid	C_29_H_50_O	45, 89, 115, 159
3	428.1944	6.24	11.26	427.1876	428.194	Lasiocarpine N‐oxide	Pyrrolizidine alkaloid	C_21_H_33_NO_8_	45, 73, 133, 341, 411
4	427.1922	6.21	11.94	426.1854	427.192	β‐amyrin	Triterpenoid	C_30_H_50_O	89, 133, 187
5	346.1478 (M + NH4^+^)	3.80	11.17	328.1144	346.148	11‐hydroperoxy‐12, 13‐epoxy‐9‐octadecenoic acid	Fatty acid	C_18_H_32_O_5_	45, 59, 87, 103, 163, 295
6	411.1588	6.36	8.55	410.1520	411.159	Lasiocarpine	Pyrrolizidine alkaloids	C_21_H_33_NO_7_	45, 99, 103, 142, 201, 243, 363
7	279.1230	4.07	8.60	278.1162	279.123	9Z, 12Z, 15E‐octadecatrienoic acid	Fatty acid	C_18_H_30_O_2_	45, 89, 103, 121, 149, 201
8	360.1634 (M + CH3OH + H+)	5.39	8.13	327.1304	360.163	Vanilic acid glucoside	Tannin	C_14_H_18_O_9_	45, 99, 279, 307
9	441.2076	6.80	8.02	440.2008	441.208	Eupatin 3‐O‐sulfate	Polyphenolic	C_18_H_16_O_11_S	99, 127, 177, 205
10	307.0778	3.17	12.57	306.0711	307.078	Cimifugin	Polyphenolic	C_16_H_18_O_6_	90, 157, 215, 259
11	447.2620	5.24	4.18	446.2552	447.262	Luteolin‐7‐O‐glucoside	Flavonoid	C_21_H_20_O_11_	89, 133, 147, 430
12	303.0834	3.82	11.18	302.0766	303.083	Methyl‐catichin	Catechin	C_16_H_16_O_6_	259
13	360.1625	4.58	5.91	359.1557	360.163	Rosamarinic acid	Phenolic	C_18_H_16_O_8_	45, 89, 147, 235, 343
14	537.5064	11.22	24.99	536.4996	537.506	Lithospermic acid	Phenolic	C_27_H_22_O_12_	75, 281, 417, 505
15	404.1885 (M + CH3OH + H+)	4.98	4.33	371.1555	404.188	7‐acetyleuropine	Pyrrolizidine alkaloid	C_18_H_29_NO_7_	45, 87, 163, 207, 353
16	374.1781	6.09	4.65	373.1714	374.178	Rosmarinic acid methyl ester	Phenolic	C_19_H_18_O_8_	73, 135, 175
17	330.1527	5.38	6.26	329.1459	330.153	Europine	Alkaloid	C_16_H_27_NO_6_	89, 131, 175
18	411.1588	6.36	8.55	410.1520	411.159	Dehydrolasiocarpine	Pyrrolizidine alkaloid	C_21_H_31_NO_7_	45, 73, 201, 275, 319
19	201.0554	3.93	19.34	200.0486	201.055	2‐Hydroxy‐3,4‐dimethoxybenzoic acid	Pyruvic acid	C_9_H_10_NO_5_	45, 93, 119
20	351.1413	4.27	5.29	350.1345	351.141	4‐(2‐Hydroxy‐3,4‐dimethoxy‐6‐methyl‐5‐(sulfooxy)phenyl)butanoic Acid	Polyphenolic	C_13_H_18_O_9_S	45, 87, 133, 157, 319
21	397.1826	7.11	13.77	396.1758	397.183	1‐Hexanol arabinosylglucoside	Polyphenolic	C_17_H_32_O_10_	87, 133, 177, 307
22	452.2061	5.28	7.48	451.1994	452.206	Rivenprost	Polyphenolic	C_24_H_34_O_6_S	73, 87, 131, 305, 435
23	496.2329	5.26	7.13	495.2261	496.233	Salvianolic acid A	Polyphenolic	C_26_H_22_O_10_	131, 219, 479
24	537.5064	11.22	24.99	536.4996	537.506	Lithospermic acid	Polyphenolic	C_27_H_22_O_12_	75, 256, 282, 417, 505
25	363.2867	11.41	9.36	362.2799	363.287	Nepetaside A	Irodide	C_17_H_30_O_8_	89, 99, 133, 195
26	331.1138	6.52	11.78	330.1070	331.114	5,8,12‐trihydroxy‐9‐octadecenoic acid	Fatty acid	C_18_H_34_O_5_	45, 98, 176, 215
27	289.1045	4.42	9.53	288.0977	289.105	9, 16‐dihydroxy‐palmitic acid	Fatty acid	C_16_H_32_O_4_	45, 87, 131, 175, 243
28	281.1382	6.01	7.79	280.1314	281.138	6E, 9E‐octadecadienoic acid	Fatty acid	C_18_H_32_O_2_	45, 87, 127, 175, 219
29	301.1832 (M + NH4^+^)	5.11	5.11	283.1499	301.183	Supinine	Pyrrolizidine alkaloid	C_15_H_25_NO_4_	45, 89, 133
30	347.1083	4.39	9.96	346.1016	347.108	Nepetaside	Irodide	C_16_H_26_O_8_	45, 89, 195
31	239.1291	4.69	7.01	238.1223	239.129	7‐angeloyl Heliotrine	Pyrrolizidine alkaloid	C_13_H_19_NO_3_	45, 89, 133
32	301.1832 (M + NH4^+^)	5.11	5.11	283.1499	301.183	3‐O‐methylgalangin	Flavonoid	C_16_H_12_O_5_	45, 89, 176
33	307.0778	3.17	12.57	306.0711	307.078	Filifolinol	Sesquiterpenoids	C_18_H_24_O_4_	45, 111, 157, 215, 259
34	403.2347	4.93	4.56	402.2280	403.235	Filifolinyl senecionate	Sesquiterpenoids	C_23_H_31_O_6_	45, 103, 133, 177, 207, 340, 386
35	289.1045	4.42	9.53	288.0977	289.105	Heliovicine	Pyrrolizidine alkaloid	C_15_H_27_NO_4_	45, 89, 131
36	295.1538 (M + CH3OH + H+)	4.54	5.46	262.1209	295.154	4, 7, 8‐trimethoxy‐naphthalene‐2‐carboxylic acid	Flavonoid	C_14_H_14_O_5_	45, 81, 201
37	301.1623	5.13	1.69	300.1555	301.162	Indicine	Pyrrolizidine alkaloid	C_15_H_25_NO_5_	41, 89, 133, 176, 284
38	375.1751 (M + Na+)	7.17	5.65	352.1864	375.175	Trichodesmine	Pyrrolizidine alkaloid	C_18_H_27_NO_6_	45, 89, 117, 174, 203
39	454.1861	5.30	6.81	453.1793	454.186	5′‐acetyllasiocarpine	Pyrrolizidine alkaloid	C_23_H_35_NO_8_	73, 87, 175
40	239.1293	5.38	4.93	238.1225	239.129	7‐Angeloylretronecine	Pyrrolizidine alkaloid	C_13_H_19_NO_3_	45, 133, 151
41	384.1980	5.69	5.26	384.1991	384.198	Punctanecine	alkaloid	C_20_H_33_NO_6_	45, 73, 103, 115, 207, 253, 321
42	448.2491	5.61	5.91	447.2423	448.249	Kaempferol‐3‐O‐glucoside	Flavonoid	C_21_H_20_O_11_	45, 131, 307, 395
43	480.2537	9.27	9.07	479.2469	480.254	Nepitrin	Flavonoid	C_22_H_22_O_12_	87, 250, 265, 357, 401
44	303.0834	3.82	11.18	302.0766	303.083	Quercetin	Flavonoid	C_15_H_10_O_7_	87, 259
45	288.1440	4.38	7.21	287.1372	288.144	luteolin	Flavonoid	C_15_H_10_O_6_	45, 59, 131, 151, 175
46	399.2339	7.26	7.89	398.2271	399.234	Echimidine	Pyrrolizidine alkaloid	C_20_H_31_NO_7_	45, 99, 133, 239, 275, 381
47	414.2445	6.68	5.10	413.2377	414.244	Echimidine N‐oxide	Pyrrolizidine alkaloid	C_20_H_31_NO_8_	45, 133, 239, 283
48	351.1413	4.27	5.29	350.1345	351.141	Erucifoline	Pyrrolizidine alkaloid	C_20_H_31_NO_8_	45, 87, 319
49	347.1083	4.39	9.96	346.1016	347.108	Europine N‐oxide	Pyrrolizidine alkaloid	C_16_H_27_NO_7_	45, 138, 201, 289, 329
50	428.2238 (M + NH4^+^)	7.19	8.65	410.1904	428.224	Squalene	Triterpenoid	C_30_H_50_	87, 99, 231, 275, 393
51	497.3689 (M + Na^+^)	10.69	14.35	474.3802	497.369	1,2‐Benzenedicarboxylic acid, diundecyl ester	Phthalic acid ester	C_30_H_50_O_4_	57, 89, 133, 199, 241
52	444.2185 (M + NH4^+^)	6.23	12.11	426.1851	444.218	Lupeol	Triterpenoid	C_30_H_50_O	45, 157, 275, 391, 409
53	400.2113	6.76	5.44	399.2046	400.211	Campesterol	Phytosterol	C_28_H_48_O	45, 87, 99, 127, 249
54	275.2371	11.45	8.13	274.2304	275.237	Naringenin	Flavonoid	C_15_H_12_O_5_	45, 57, 85, 107
55	448.2131 (M + CH_3_OH + H^+^)	5.32	4.88	415.1801	448.213	‐sitosterol	Steroid	C_25_H_50_O	45, 89, 163, 397
56	686.3633	9.42	12.20	685.3565	686.363	Citbismine C	Acridone	C_37_H_36_N_2_O_11_	89, 99, 133, 177, 360, 533
57	293.0994	4.38	7.36	292.0926}	293.099	Lycoricidine	Phenanthridine	C_14_H_13_NO_6_	45, 127, 245
58	289.1581	10.52	7.58	288.1513	289.158	5,7,2′,5′ ‐Tetrahydroxy flavanone	Flavonoid	C_15_H_12_O_6_	43, 57, 69, 73, 150, 201
59	325.1265	4.79	5.82	324.1198	325.127	Acremoauxin A	Alkaloid	C_16_H_21_NO_6_	45, 73, 103, 159
60	337.1265	4.36	5.66	336.1197	337.126	Semilepidinoside A	Glycoside	C_16_H_20_N_2_O_6_	45, 89, 191
61	263.1284	6.02	8.04	262.1216	263.128	8‐deoxylactucin	Lactones	C_15_H_16_O_4_	45, 55, 99, 133, 157
62	372.2018	5.71	8.47	371.1950	372.202	Asplenetin	Flavonoid	C_20_H_20_O_7_	45, 89, 177,283
63	388.2290 (M + NH4^+^)	5.73	4.84	370.1957	388.229	Benzoic acid, 2,4‐bis[(trimethylsilyl)oxy]‐, trimethylsilyl ester	Benzoic acid derivative	C_16_H_30_O_4_Si_3_	45, 89, 133, 177, 327
64	191.0737	4.90	5.32	190.0669	191.074	7‐Methoxy‐4‐methylcoumarin	Coumarins	C_11_H_10_O_3_	31, 45, 59, 103, 173
65	291.1588	6.95	7.05	290.1520	291.159	Epicatechin	Flavonoid	C_15_H_14_O_6_	45, 59, 87, 141, 163, 243
66	400.1930	5.08	8.60	399.1862	400.193	Heliosupine	Alkaloid	C_20_H_31_NO_6_	45, 89, 159, 189, 247
67	516.2741	7.15	8.30	515.2673	516.274	4,5‐di‐O‐cafeoylquinic acid	Phenolic acid	C_25_H_24_O_12_	73, 133, 231, 279, 407, 481
68	339.1787	5.80	5.15	338.1720	339.179	3‐O‐p‐coumaryl quinic acid	Phenolic acid	C_16_H_18_O_8_	45, 70, 133, 140, 172, 322
69	386.1784 (M + NH4^+^)	5.85	6.58	368.1451	386.178	3‐O‐feruloyl quinic acid	Phenolic acid	C_17_H_20_O_9_	45, 103, 147, 207, 333
70	365.1938	7.42	8.05	364.1870	365.194	Senkirkine	Pyrrolizidine alkaloid	C_19_H_27_NO_6_	45, 73, 87, 99, 133, 275
71	186.1313	8.28	8.55	185.1245	186.131	Otonecine	Pyrrolizidine alkaloid	C_9_H_15_NO_3_	41, 55, 71, 154
72	357.2135 (M + NH4^+^)	7.09	8.18	339.1802	357.213	Tetracosane	Essential oil	C_24_H_50_	45, 69, 87
73	413.2127	6.69	9.03	412.2059	413.213	Stigmasterol	Terpenoid	C_29_H_48_O	45, 89, 133, 245, 395
74	407.1641 (M + Na^+^)	4.93	4.33	384.1754	407.164	2,3‐Butanediol apiosylglucoside	glycoside	C_15_H_28_O11	87, 175, 350
75	436.1768	5.37	6.55	435.1700	436.177	6′‐Hydroxymethyl simvastatin	Lactone	C_25_H_38_O_6_	45, 87, 99, 173, 189, 235, 391
76	368.2041	6.87	6.96	367.1973	368.204	4‐Acetyl‐2‐prenylphenol glycoside	Glycoside	C_19_H_26_O_7_	67, 139, 229, 333
77	201.0912	5.11	13.04	200.0844	201.091	Dihydroclavaminic acid	Monocarboxylic acid	C_8_H_12_N_2_O_4_	43, 141
78	335.1717	6.87	5.65	334.1649	335.172	Senecionine	Pyrrolizidine alkaloid	C1_8_H_25_NO_5_	45, 69, 133, 195, 303, 317
79	332.1686 (M + NH4^+^)	4.84	5.77	314.1353	332.169	Isorhamnetin	Flavonoid	C_16_H_12_O_7_	89, 175, 195, 219
80	578.3089	5.97	5.23	577.3022	578.309	Procyanidin B1	Flavonoid	C_30_H_26_O_12_	89, 103, 133, 367, 543, 562
81	370.2194 (M + NH4^+^)	6.94	4.92	352.1861	370.219	Corynanthine	Alkaloid	C_21_H_26_N_2_O_3_	45, 131, 239
82	391.2595	10.96	14.77	390.2527	391.259	Diisooctyl phthalate	Benzoic acid derivative	C_24_H_38_O_4_	71, 149, 167
83	293.1751	6.44	4.56	292.1683	293.175	9,12,15‐Octadecatrienoic acid, methyl ester, (Z,Z,Z)—	Fatty acid	C_19_H_32_O_2_	45, 89, 99, 121, 178
84	332.2696	11.30	12.60	331.2628	332.270	Hexadecanoic acid,2,3‐dihydroxypropyl ester	Fatty acid	C_19_H_38_O_4_	58, 91, 98, 240
85	281.1017 (M + CH3OH + H+)	3.80	11.29	248.0687	281.102	Benzene, (1‐ethyldecyl)	Aromatic hydrocarbon	C_18_H_30_	45, 73, 99, 117, 203
86	387.3399	10.85	9.81	386.3331	387.340	Cholesterol	Steroid	C_27_H_46_O	70, 83, 161, 203, 337
87	460.2134	5.68	9.93	459.2066	460.213	Lankacidin C	Polyketide	C_25_H_33_NO_7_	87, 103, 191, 235, 425
88	347.2921	10.86	16.25	346.2853	347.292	Nepetaside	Irodide	C_16_H_26_O_8_	45, 89, 109, 151, 315,330
89	364.3179	10.87	15.13	363.3111	364.318	Nepetaside A	Irodide	C_17_H_30_O_8_	45, 85, 151
90	293.0994	4.38	7.36	292.0926	293.099	Carbinoxamine	Carbamate	C_16_H_19_ClN_2_O	45, 127, 155, 201, 245
91	572.2986	8.33	7.93	571.2918	572.299	Endomorphin‐2	Peptide	C_32_H_37_N_5_O_5_	89. 99, 133, 221, 449, 537, 555

**TABLE 6:** Binding free energy and inhibition constants of compounds in complex with respective proteins.

Compounds	AChE		BChE		α‐amylase		α‐glucosidase		Tyrosinase	
	Free energy of binding, kcal/mol	Estimated inhibition constant	Free energy of binding, kcal/mol	Estimated inhibition Constant	Free energy of binding, kcal/mol	Estimated inhibition constant	Free energy of binding, kcal/mol	Estimated inhibition constant	Free energy of binding, kcal/mol	Estimated inhibition constant
Known inhibitors^a^	−9.27	159.75 nM	−8.17	1.02 μM	−10.66	15.33 nM	−10.31	27.69 nM	−6.44	18.98 μM
Rosmarinic acid	−17.21	244.49 fM	−17.41	172.06 fM	−13.98	56.79 nM	13.97	57.55 pM	−13.78	78.95 pM
Lithospermic acid	−11.18	6.37 nM	13.39	153.21 pM	−9.14	199.93 nM	−10.10	39.58 nM	−9.94	51.50 nM
Nepetaside A	−15.90	2.21 pM	15.13	8.19 Pm	−12.12	1.31 nM	−13.20	210.63 pM	−11.45	4.04 nM
9, 16‐dihydroxy‐palmitic acid	−6.89	8.93 μM	−6.14	31.61 μM	−3.42	3.10 mM	−5.61	77.14 μM	−6.72	11.77 μM
Filifolinol	−8.83	336.17 nM	−8.23	925.84 nM	−7.18	5.46 μM	−7.79	1.95 μM	−6.19	28.94 μM
Heliovicine	−9.49	110.46 nM	−9.11	208.69 nM	−9.27	159.85 nM	−9.94	52.18 nM	−7.43	3.59 μM
Indicine	−11.12	7.01 nM	−9.90	55.12 nM	−9.58	95.58 nM	−10.60	16.87 nM	−8.75	383.07 nM
Trichodesmine	−13.15	230.65 pM	13.32	171.61 pM	−11.71	2.62 nM	−11.33	4.99 nM	−10.35	25.72 nM
5′‐acetyllasiocarpine	−12.18	1.19 nM	−11.09	7.45 nM	−8.54	553.98 nM	−7.57	2.82 μM	−7.73	2.17 μM
7‐Angeloylretronecine	−7.56	−2.87 μM	−6.96	7.90 μM	−7.56	2.87 μM	−8.29	835.13 nM	−6.05	36.45 μM
Echimidine N‐oxide	−12.91	341.49 pM	−11.37	4.60 nM	−9.90	55.05 nM	−10.42	23.19 nM	−9.19	184.05 nM
Europine N‐oxide	−10.36	25.50 nM	−9.99	47.95 nM	−10.15	36.23 nM	10.50	20.16 nM	−8.08	1.19 μM
4,5‐di‐O‐cafeoylquinic acid	−14.94	11.24 pM	14.43	26.30 pM	−10.10	39.81 nM	−10.88	10.62 nM	−11.23	5.87 nM

^a^ For α‐glucosidase and α‐amylase, acarbose is used as reference compound. Galantamine is used reference compound for AChE and BChE. While kojic acid is used as reference for Tyrosinase.

**TABLE 7:** Details of Interaction Patterns after Post‐Dock Analysis of Docked Complex for Selected Compounds.

Ligands	Bond category	Bond distance	Bond type	Interactions			
				Residue name and groups	From chemistry	Residue name and groups	To chemistry
**AChE**							
Nepetaside A	Hydrogen Bond	2.99577 2.21249 2.42639	Conventional Hydrogen Bond	A:TYR337:HH :LIG1:H :LIG1:H	H‐Donor	:LIG1:O A:TYR72:O A:TYR124:OH	H‐Acceptor
	Hydrogen Bond	2.90964 2.93914	Carbon Hydrogen Bond	:LIG1:C :LIG1:C	H‐Donor	A:TYR133:OH A:GLU202:OE1	H‐Acceptor
	Hydrophobic	5.39925 4.34928 4.39488 4.98013	Pi‐Alkyl	A:TRP86 A:TYR337 A:PHE338 A:HIS447	Pi‐Orbitals	:LIG1:C :LIG1:C :LIG1:C :LIG1:C	Alkyl
Rosmarinic acid	Hydrogen Bond	2.7884 2.52487 2.02476 1.81424 1.74844 2.03323	Conventional Hydrogen Bond	A:SER125:HG A:ARG296:HN :LIG0:H :LIG0:H :LIG0:H :LIG0:H	H‐Donor	:LIG0:O :LIG0:O A:HIS447:O A:HIS447:O A:SER293:O A:SER293:O	H‐Acceptor
	Electrostatic	4.65502	Pi‐Cation	A:HIS447:NE2	Positive	:LIG0	Pi‐Orbitals
	Hydrogen Bond	2.92855	Pi‐Donor Hydrogen Bond	A:PHE295:HN	H‐Donor	:LIG0	Pi‐Orbitals
	Hydrophobic	3.78526	Pi‐Sigma	A:HIS447:CD2	C‐H	:LIG0	Pi‐Orbitals
	Hydrophobic	5.0499 5.11088	Pi‐Pi T‐shaped	A:TRP86 A:TRP86	Pi‐Orbitals	:LIG0 :LIG0	Pi‐Orbitals
**BChE**							
Nepetaside A	Hydrogen Bond	2.18382 2.83102 2.21441 1.88216 1.76565	Conventional Hydrogen Bond	: A:SER198:HG A:HIS438:HE2 :LIG1:H :LIG1:H :LIG1:H	H‐Donor	:LIG1:O :LIG1:O A:HIS438:O A:GLU197:OE1 :LIG1:O	H‐Acceptor
	Hydrogen Bond	3.07455 2.74839 3.64317	Carbon Hydrogen Bond	A:TRP82:CD1 A:HIS438:CD2 :LIG1:C	H‐Donor	:LIG1:O :LIG1:O A:GLY78:O	H‐Acceptor

	Hydrophobic	3.37775	Pi‐Sigma	:LIG1:C	C‐H	A:TYR332	Pi‐Orbitals
	Hydrophobic	5.2423	Alkyl	A:ALA328	Alkyl	:LIG1	A:ALA328
Hydrophobic	5.47067 4.05101 4.49939	Pi‐Alkyl	A:PHE329 A:PHE329 A:TYR332	Pi‐Orbitals	:LIG1 :LIG1:C :LIG1:C	Alkyl
Rosmarinic acid	Hydrogen Bond	2.45621 2.13739 2.30329 2.34102 2.43742	Conventional Hydrogen Bond	A:GLY121:HN A:SER198:HG :LIG0:H :LIG0:H :LIG0:H	H‐Donor	:LIG0:O :LIG0:O A:GLU197:OE1 A:ASP70:OD1 A:ASP70:OD1	H‐Acceptor
	Hydrogen Bond	2.98552 2.93862	Carbon Hydrogen Bond	A:SER198:CB A:GLY439:CA	H‐Donor	:LIG0:O :LIG0:O	H‐Acceptor
	Hydrophobic	3.57957	Pi‐Sigma	A:TRP82:CB	C‐H	:LIG0	Pi‐Orbitals
	Hydrophobic	5.17868 5.37393	Pi‐Pi Stacked	A:TRP82 A:TRP82	Pi‐Orbitals	:LIG0 :LIG0	Pi‐Orbitals
**Amylase**							
Nepetaside A	Hydrogen Bond	2.33924 2.70956 3.08736 1.96834 2.56627	Water Hydrogen Bond;Carbon Hydrogen Bond	:LIG1:H :LIG1:H :LIG1:H :LIG1:H :LIG1:H	H‐Donor	A:HOH563:O A:HOH527:O A:HOH537:O A:HOH527:O A:HOH537:O	H‐Acceptor
	Hydrogen Bond	2.24905	Conventional Hydrogen Bond	A:ARG344:HH11	H‐Donor	:LIG1:O	H‐Acceptor
	Hydrogen Bond	2.802	Carbon Hydrogen Bond	A:HIS80:CD2	H‐Donor	:LIG1:O	H‐Acceptor
	Hydrophobic	4.1395 4.66682	Alkyl	A:LEU166 :LIG1:C	Alkyl	:LIG1 A:LEU166	Alkyl
Rosmarinic acid	Hydrogen Bond	2.06339 1.84707 2.1709 1.87478	Carbon Hydrogen Bond	:LIG0:H :LIG0:H :LIG0:H :LIG0:H	H‐Donor	A:HOH653:O A:HOH653:O A:HOH513:O A:HOH513:O	H‐Acceptor

	Hydrogen Bond	2.62515 2.15589	Conventional Hydrogen Bond	A:ARG344:HH11 A:ARG344:HH21	H‐Donor	:LIG0:O :LIG0:O	H‐Acceptor
	Hydrophobic	3.11629	Carbon Hydrogen Bond	A:THR207:CA	H‐Donor	:LIG0:O	H‐Acceptor
	Electrostatic	4.08453	Pi‐Anion	A:GLU230:OE2	Negative	:LIG0	Pi‐Orbitals
Hydrophobic	5.48087	Pi‐Alkyl	:LIG0	Pi‐Orbitals	A:LEU166	Alkyl
**Glucosidase**							
Nepetaside A	Hydrogen Bond	3.77719 3.22562	Carbon Hydrogen Bond	:LIG1:C :LIG1:C	H‐Donor	A:ASN230:O A:SER505:O	H‐Acceptor
	Hydrophobic	4.6773 4.93867 4.44816 4.00038 3.80329 4.4394	Alkyl	A:ALA231 A:ILE233 A:LYS506 A:LYS506 :LIG1:C :LIG1:C	Alkyl	:LIG1 :LIG1 :LIG1 :LIG1 A:LYS506 A:ILE233	Alkyl
Rosmarinic acid	Hydrogen Bond	2.23994 2.02921 2.0974 2.4219	Conventional Hydrogen Bond	A:ASN475:HN :LIG0:H :LIG0:H :LIG0:H	H‐Donor	:LIG0:O A:ASP232:OD1 A:LYS506:O A:ASN237:OD1	H‐Acceptor
	Electrostatic	3.02752	Pi‐Anion	A:ASP232:OD2	Negative	:LIG0	Pi‐Orbitals
	Hydrophobic	4.8063 4.80563 5.2179	Pi‐Alkyl	:LIG0 :LIG0 :LIG0	Pi‐Orbitals	A:LYS506 A:ILE233 A:ALA234	Alkyl
**Tyrosinase**							
Nepetaside A	Hydrogen Bond	2.2847	Conventional Hydrogen Bond	:LIG1:H	H‐Donor	A:MET215:O	H‐Acceptor
	Hydrophobic	5.48583 4.07607	Alkyl	A:PRO201 :LIG1:C	Alkyl	:LIG1 A:PRO201	Alkyl
	Hydrophobic	5.17531 4.79015	Pi‐Alkyl	A:PHE197 A:PHE197	Pi‐Orbitals	:LIG1 :LIG1:C	Alkyl
Rosmarinic acid	Hydrogen Bond	2.13378 2.91906 2.92274 2.51815	Conventional Hydrogen Bond	A:ASN205:HD21 A:HIS208:HD1 :LIG0:H :LIG0:H	H‐Donor	:LIG0:O :LIG0:O A:GLU195:OE1 A:VAL217:O	H‐Acceptor
	Hydrophobic	3.81053 3.79838	Pi‐Sigma	A:MET61:ce A:VAL218:CG2	C‐H	:LIG0 :LIG0	Pi‐Orbitals
	Hydrophobic	5.01629 3.56368	Pi‐Pi Stacked	A:PHE197 A:HIS208	Pi‐Orbitals	:LIG0 :LIG0	Pi‐Orbitals
	Hydrophobic	4.74199	Pi‐Pi T‐shaped	:LIG0	Pi‐Orbitals	:LIG0	Pi‐Orbitals
	Hydrophobic	4.97825 3.90908 4.61016	Pi‐Alkyl	:LIG0 :LIG0 :LIG0	Pi‐Orbitals	A:ALA221 A:CU501:Cu A:CU502:Cu	Alkyl

#### 3.5.2 Tyrosinase Inhibition Activities

Tyrosinase inhibitory activity was measured as kojic acid equivalent (Table 4), where HE‐M, HE‐E, and HE‐W fractions showed nearly equal inhibition with values of 47.63 ± 1.43, 47.16 ± 0.90, and 47.79 ± 2.90 mg KAE/g extract, respectively; whereas, the HE‐H fraction exhibited inhibitory potential of 41.40 ± 1.15 mg KAE/g extract. These observations revealed that the anti‐tyrosinase inhibitory activities of all the fractions of *H. europaeum* can be attributed to the synergic effects of a variety of secondary metabolites, which make *H. europaeum* or its crude extract a significant agent for skin care products.

#### 3.5.3 α‐Amylase and α‐Glucosidase Inhibitory Activities

Diabetes is a chronic disease that leads to high mortality risk, and thus has a bad impact on the economy and normal everyday life. Some drugs are available in the market, but one or more side effects are associated with these allopathic drugs (Osadebe et al. 2014). The safer way to combat diabetes is to use an alternative medicine system. Several plants have been reported to afford antidiabetic properties and may directly affect insulin secretion; that's why the use of natural plant products is increasing in the alternative medicine system (Upadhyay 2016). Further, the World Health Organization (WHO) supports and encourages the use of herbal medicine as anti‐diabetic drugs (Bailey and Day 1989). Previously, plant sources have been used to lower blood glucose in experimental models and are identified as useful in discovering safer anti‐diabetic drugs, for example, the α‐glucosidase inhibitor acarbose and galegine, which contributed to (L Harvey 2010). Thus, carbohydrate hydrolyzing enzymes α‐amylase and α‐glucosidase are the best therapeutic targets to study natural products as anti‐diabetic agents.

All the extracts of *H. europaeum* were tested for their α‐amylase and α‐glucosidase inhibitory potential as acarbose equivalent (Table 4). HE‐H and HE‐E exhibited almost equal anti‐α‐amylase potential with the values of 0.43 ± 0.01 and 0.42 ± 0.01 mmol ACAE/g extract, respectively, while in α‐glucosidase inhibitory assay, interestingly, the HE‐H fraction was found to be active; however, HE‐M, HE‐E, and HE‐W displayed inhibitory values of 1.77 ± 0.00, 1.77 ± 0.04, and 1.71 ± 0.02 mmol ACAE/g extract, respectively. These results unveiled the anti‐diabetic properties of *H. europaeum* and offer its candidature to be considered for further studies and may be utilized in antidiabetic alternative medicine. Previous studies disclosed that in a rat model, *H. strigosum* and *H. indicum* have shown significant antidiabetic activity (Chaudhry et al. 2016; Ibrahim et al. 2016) also, a crude extract of *Heliotropium curassavicum exhibited antidiabetic effects* (Akbar et al. 2023 *). Our findings have identified* a *new source with antidiabetic potential*.

### 3.6 Multivariate Analysis

**FIGURE 1:**
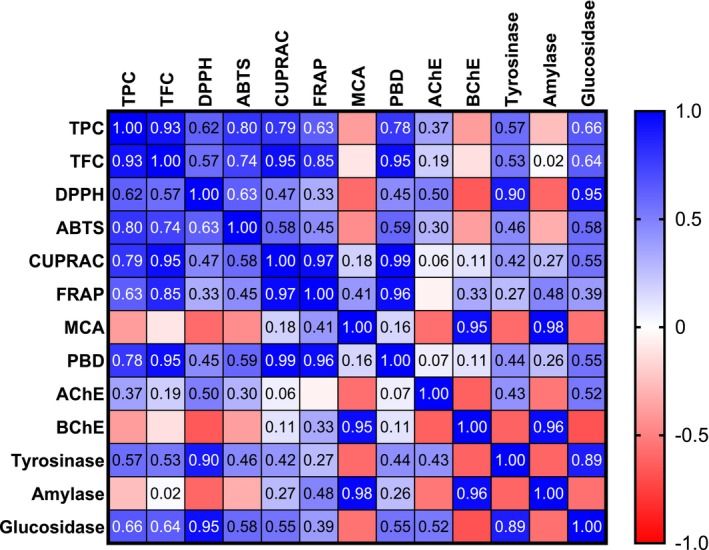
Pearson correlation values between biological activity assays (*p* < 0.05). TPC, total phenolic content; TFC, total flavonoid content; ABTS, 2,2′‐azino‐bis(3‐ethylbenzothiazoline‐6‐sulphonic acid; DPPH, 1,1‐diphenyl‐2‐picrylhydrazyl; CUPRAC, cupric reducing antioxidant capacity; FRAP, ferric reducing antioxidant power; MCA, metal chelating assay; PBD, phosphomolybdenum; AChE, acetylcholinesterase; BChE, butyrylcholinesterase.

Based on the antioxidant and enzyme inhibition tests, we performed a correlation analysis and the results are shown in Figure 1. Apparently, the antioxidant properties were strongly correlated with the total phenolics/flavonoids content, except for the metal chelating effect. According to the correlation values (*R* > 0.8), flavonoids in particular were key players in the reducing power tests (CUPRAC and FRAP). Consistent with our findings, several researchers reported a linear correlation between total phenolics/flavonoids content and antioxidant properties (Hasanagić et al. 2024; He et al. 2025). The position and number of hydroxyl groups in the phenolic rings may explain their antioxidant properties. However, the observed metal chelating activity can be explained by the presence of nonphenolic chelators, including polysaccharides or peptides. Regarding the enzyme inhibitory effects, the observed AChE and BChE inhibitory effects did not correlate with the total bioactive components. In this sense, the observed effects may be associated with the presence of nonphenolic inhibitors such as alkaloids. However, the inhibitory effect of glucosidase and tyrosinase correlated moderately with the total bioactive compounds. In particular, from Table 4, some flavonoids can be attributed to the observed glucosidase and tyrosinase inhibitory effects (Carrillo‐Martinez et al. 2024; Nezhad Salari et al. 2024; Xu et al. 2024). However, we strongly recommend carrying out the isolation process of the compounds in the tested extracts and evaluating their enzyme inhibitory effects individually.

**FIGURE 2:**
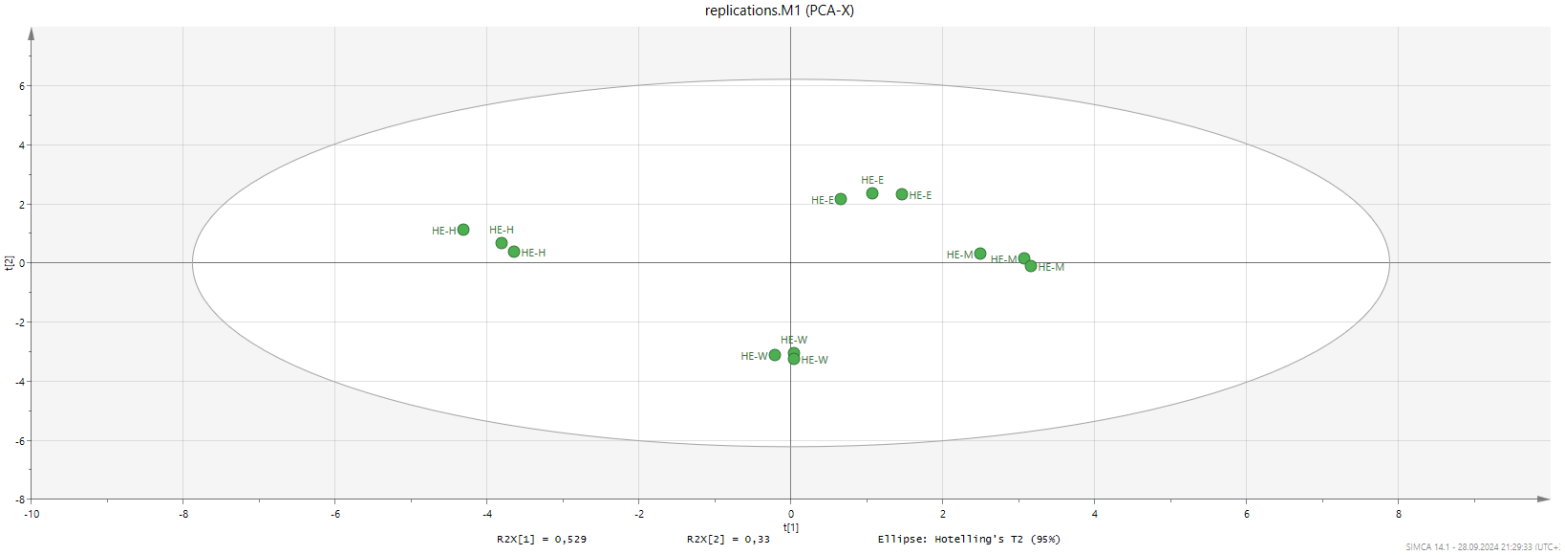
Principal component analysis between tested samples and biological activity assays.

To understand the differences between the tested extracts and fractions, we performed principal component analysis. The results are shown in Figure 2. Two components (PC1: 53% and PC2: 33%) account for 86% of the total components. Based on the PCA diagram, the extracts can be divided into three groups. The n‐hexane and water extracts were divided into individual groups. However, the ethanol and methanol extracts belonged to the same group. In particular, the n‐hexane extract did not contain any flavonoids and had the weakest effect in antioxidant tests, as did the water extract. Thus, the n‐hexane and water extracts were far away from the ethanol and methanol extracts. When evaluating all biological activity results, ethanol and methanol may be useful as solvents for the production of health‐promoting applications using *H. europaeum* .

### 3.7 Post Dock Analysis

In order to find the best binding pose for each protein, the conformation having the lowest rmsd clustering and binding free energy was identified. The binding free energies and estimated inhibition constants for each protein‐ligand complex were clustered in Table 6

The lowest binding free energy was observed for 9, 16‐dihydroxy‐palmitic acid against α‐amylase i.e., −3.42 kcal/mol. The highest was obtained for Rosmarinic acid against AChE and BChE i.e., 17.21 and 17.41 kcal/mol, respectively. The ranges of estimated binding energies were found to be −6.89 to −17.21 kcal/mol for AChE, for BChE the range was −6.14 to −17.41 kcal/mol, for α‐amylase −3.42 to −13.98 kcal/mol, for α‐glucosidase −5.61 to −13.97 kcal/mol, and for tyrosinase it was found to be −6.05 to −13.78 kcal/mol.

**FIGURE 3:**
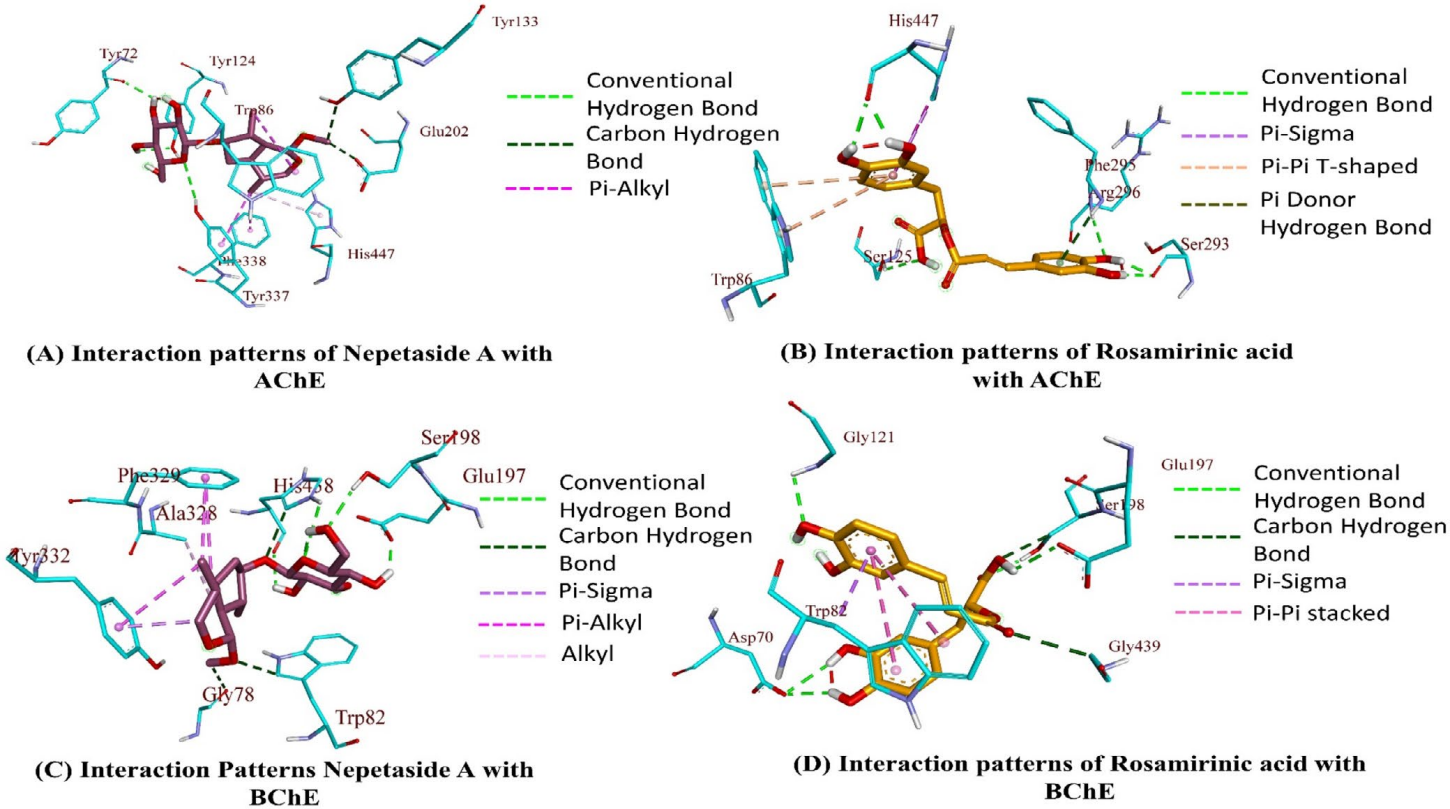
Binding interactions of compounds with AChE and BChE.

**FIGURE 4:**
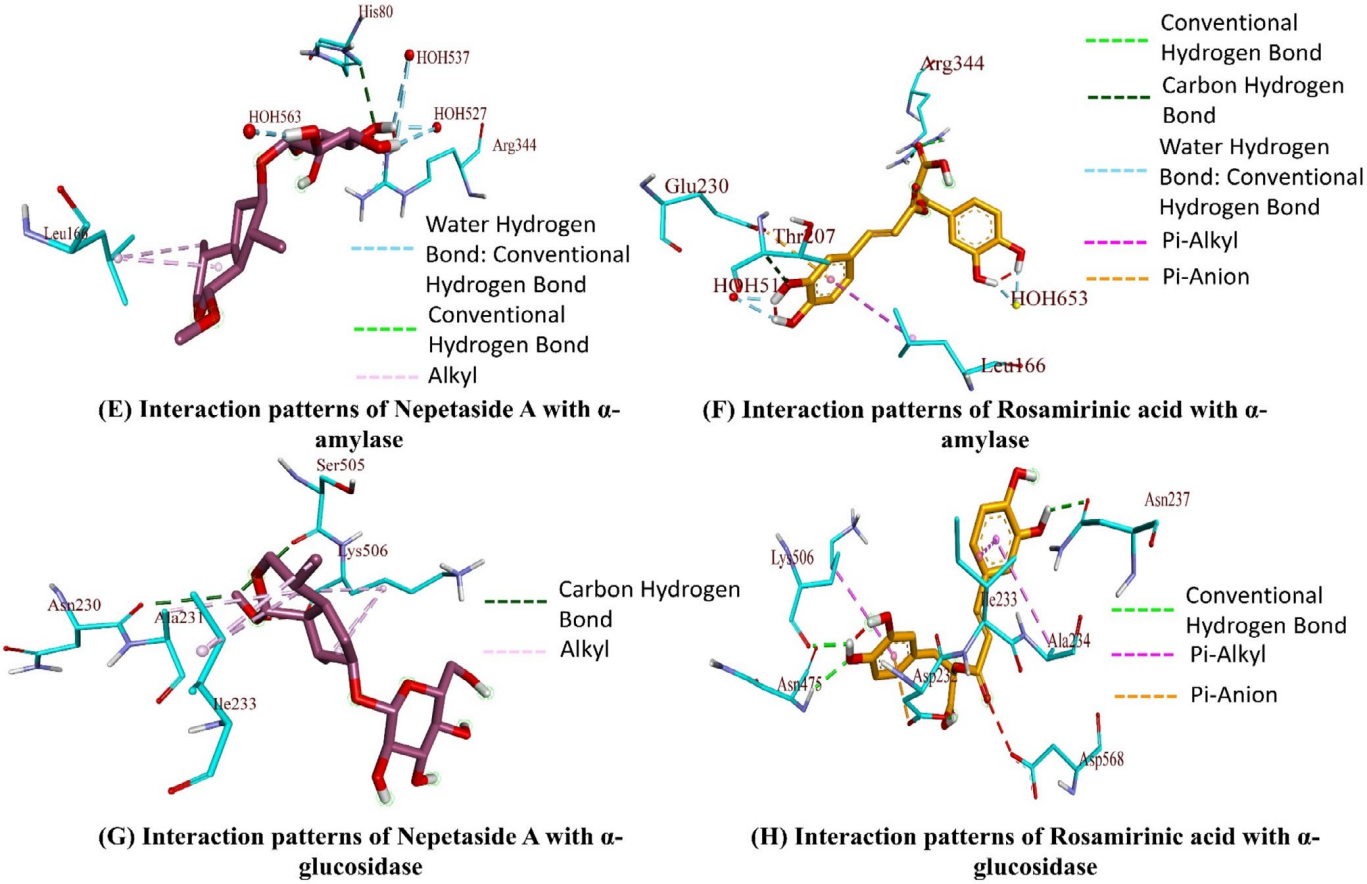
Binding interactions of compounds with α‐amylase and α‐glucosidase.

**FIGURE 5:**
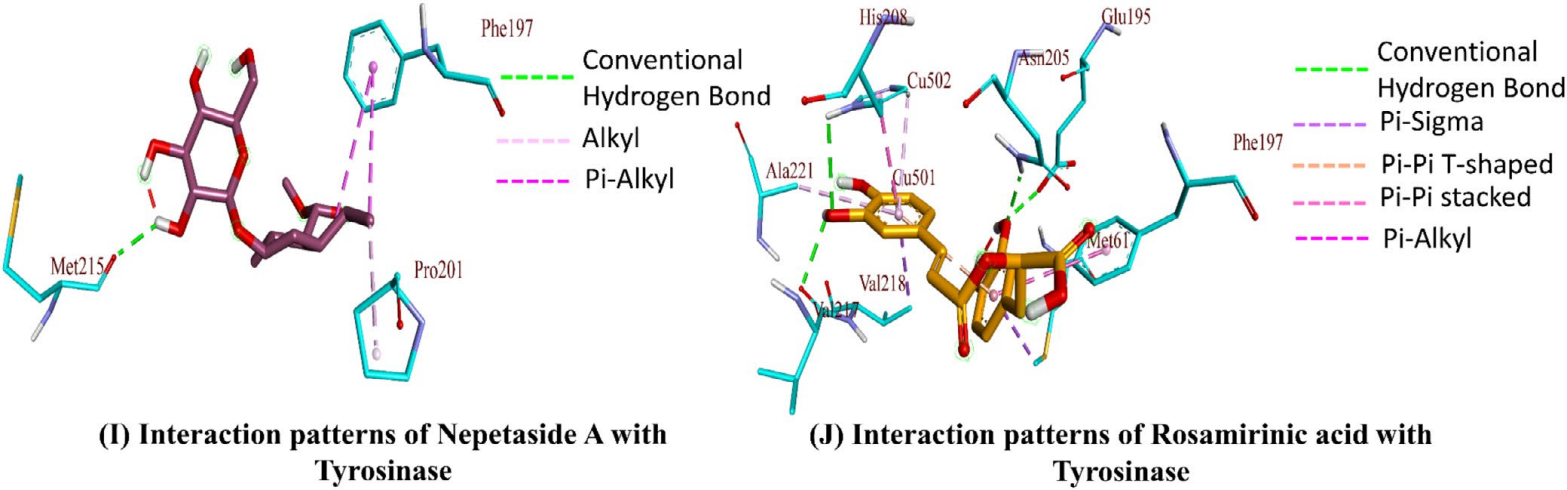
Binding interactions of compounds with tyrosinase.

The binding affinities for Rosmarinic acid and Nepetaside A were found to be higher than the rest of the compounds against all five enzymes. The post‐dock analysis of these two compounds against the five enzymes showed that these two compounds show significant hydrogen bonds along with hydrophobic interactions (Figures 3, 4, 5). Table 7 summarizes the interaction patterns for the compounds with their respective enzymes. the compound 9,16‐dihydroxy‐palmitic acid was found to be least active against BChE, AChE, α‐glucosidase, and α‐amylase, while 7‐Angeloylretronecine was found to have the least binding energy against tyrosinase, then the rest of the compounds; however, the binding free energy is still comparable to control kojic acid.

The binding affinity of rosmarinic acid was found to be −13.78 kcal/mol against AChE with multiple hydrogen bonds, electrostatic, and hydrophobic interactions (Figure 3B). The binding affinity of the BChE –rosmarinic acid complex was observed to be −17.21 kcal/mol with multiple hydrogen bonds and various hydrophobic interactions (Figure 3D). The binding affinity for α‐glucosidase‐ rosmarinic acid complex was −17.41 kcal/mol with four hydrogen bonds and some hydrophobic interactions (Figure 4F). The binding affinity of α‐amylase –rosmarinic acid complex was −13.98 kcal/mol with multiple hydrogen bonds, electrostatic, and hydrophobic interactions (Figure 4H). The binding affinity of the tyrosinase –rosmarinic complex was raised to 13.97 kcal/mol with hydrogen bonds and hydrophobic interactions (Figure 5J). The binding affinities of nepetaside A were found to be −11.45, −15.90, −15.13, −12.12, and −13.20 kcal/mol against Ache, BChE, α‐glucosidase, α‐amylase, and tyrosinase respectively, with nepetaside A mediating hydrogen bonds and hydrophobic interactions with all five enzymes (Figures 3, 4, 5).

## 4 Conclusion

Methanolic extract of *H. europaeum* was divided into hexane, ethyl acetate, and water soluble fractions, which were analyzed for their secondary metabolic contents and bioactivities. Overall, crude methanolic extract was found to be rich in alkaloids, phenolics, flavonoids, terpenoids, steroids, and glycosides, and also showed a significant level of antioxidant and enzyme inhibitory potential. These results were further verified by docking studies and multivalent analysis. Although this provides clear evidence that plants of the *Heliotropium* genus have several medicinal importance in the treatment of diverse diseases. The medicinal use of these plants as phyto‐pharmaceuticals would depend on the production of the required systematic procedures necessary to standardize the various bioactive secondary metabolites in such herbal formulations. The second important factor regarding the use of *H. europaeum* in herbal pharmaceutical preparation is to avoid contamination by seeds of this plant. Therefore, we conclude that in the coming times, plants of the *Heliotropium* genus would become an acceptable source of indigenous medicines (Figures 3, 4, 5).

## Author Contributions

**Shabnam Mustafa:** conceptualization (equal), data curation (equal), investigation (equal), methodology (equal), writing – original draft (equal), writing – review and editing (equal). **Muhammad Imran Tousif:** conceptualization (equal), funding acquisition (equal), investigation (equal), methodology (equal), supervision (equal), writing – original draft (equal), writing – review and editing (equal). **Naheed Raiz:** data curation (equal), investigation (equal), methodology (equal), writing – original draft (equal). **Muhammad Saleem:** data curation (equal), investigation (equal), methodology (equal), validation (equal), writing – original draft (equal). **Saba Tauseef:** formal analysis (equal), investigation (equal), methodology (equal), supervision (equal), writing – original draft (equal). **Gokhan Zengin:** conceptualization (equal), data curation (equal), investigation (equal), methodology (equal), writing – original draft (equal), writing – review and editing (equal). **Laiba Hassan:** conceptualization (equal), investigation (equal), software (equal), visualization (equal), writing – original draft (equal). **Areeba Hassan:** data curation (equal), investigation (equal), writing – original draft (equal), writing – review and editing (equal). **Mamona Nazir:** investigation (equal), supervision (equal), writing – original draft (equal), writing – review and editing (equal). **Shabbir Muhammad:** investigation (equal), methodology (equal), supervision (equal), writing – original draft (equal), writing – review and editing (equal).

## Ethics Statement

The authors have nothing to report.

## Consent

The authors have nothing to report.

## Conflicts of Interest

The authors declare no conflicts of interest.

## Data Availability

Data will be made available on request.
